# Metronomic Doses of Temozolomide Enhance the Efficacy of Carbon Nanotube CpG Immunotherapy in an Invasive Glioma Model

**DOI:** 10.1371/journal.pone.0148139

**Published:** 2016-02-01

**Authors:** Mao Ouyang, Ethan E. White, Hui Ren, Qin Guo, Ian Zhang, Hang Gao, Song Yanyan, Xuebo Chen, Yiming Weng, Anna Da Fonseca, Sunny Shah, Edwin R. Manuel, Leying Zhang, Steven L. Vonderfecht, Darya Alizadeh, Jacob M. Berlin, Behnam Badie

**Affiliations:** 1 Department of Cardiology, Third Xiangya Hospital, Central South University, Changsha Hunan, P.R. China; 2 Irell & Manella Graduate School of Biological Sciences at City of Hope, Duarte, California, 91010, United States of America; 3 Department of Molecular Medicine, City of Hope Beckman Research Institute, Duarte, California, 91010, United States of America; 4 Department of General Surgery, The Second Hospital of Jilin University, Changchun, Jilin Province, P.R. China; 5 Department of Gastroenterology, Third Xiangya Hospital, Central South University, Changsha Hunan, P.R. China; 6 Division of Neurosurgery, City of Hope Beckman Research Institute, Duarte, California, 91010, United States of America; 7 Department of Bone and Joint Surgery, No.1 Hospital of Jilin University, Changchun, Jilin Province, P.R. China; 8 Department of Nephrology, The Second Hospital of Jilin University, Changchun, Jilin Province, P.R. China; 9 Department of General Surgery, China Japan Union Hospital of Jilin University, Changchun, Jilin Province, P.R. China; 10 Laboratório de Morfogênese Celular, Instituto de Ciências Biomédicas, Universidade Federal do Rio de Janeiro, Rio de Janeiro, Brazil; 11 Division of Translational Vaccine Research, Department of Virology, City of Hope Beckman Research Institute, Duarte, California, 91010, United States of America; 12 Division of Comparative Medicine, City of Hope Beckman Research Institute, Duarte, California, 91010, United States of America; 13 Department of Cancer Immunotherapeutics & Tumor Immunology City of Hope Beckman Research Institute, Duarte, California, 91010, United States of America; Swedish Neuroscience Institute, UNITED STATES

## Abstract

Even when treated with aggressive current therapies, most patients with glioblastoma survive less than two years. Rapid tumor growth, an invasive nature, and the blood-brain barrier, which limits the penetration of large molecules into the brain, all contribute to the poor tumor response associated with conventional therapies. Immunotherapy has emerged as a therapeutic approach that may overcome these challenges. We recently reported that single-walled carbon nanotubes (SWCNTs) can be used to dramatically increase the immunotherapeutic efficacy of CpG oligonucleotides in a mouse model of glioma. Following implantation in the mouse brain, the tumor cell line used in these previous studies (GL261) tends to form a spherical tumor with limited invasion into healthy brain. In order to evaluate SWCNT/CpG therapy under more clinically-relevant conditions, here we report the treatment of a more invasive mouse glioma model (K-Luc) that better recapitulates human disease. In addition, a CpG sequence previously tested in humans was used to formulate the SWCNT/CpG which was combined with temozolomide, the standard of care chemotherapy for glioblastoma patients. We found that, following two intracranial administrations, SWCNT/CpG is well-tolerated and improves the survival of mice bearing invasive gliomas. Interestingly, the efficacy of SWCNT/CpG was enhanced when combined with temozolomide. This enhanced anti-tumor efficacy was correlated to an increase of tumor-specific cytotoxic activity in splenocytes. These results reinforce the emerging understanding that immunotherapy can be enhanced by combining it with chemotherapy and support the continued development of SWCNT/CpG.

## Introduction

Glioblastoma is the most common and most malignant form of primary brain cancer in humans. Even when treated with surgery, radiation, and chemotherapy, most patients with glioblastoma survive less than two years [1]. Although immunotherapy is being studied as a potential treatment, the local immunosuppressive tumor milieu is thought to be a major contributor to the poor responsiveness of brain tumors in many immunotherapies [2]. Tumor-induced production of immunosuppressive factors, reduced expression of MHC class I molecules by tumors, and a scarcity of antigen presenting cells are all factors that contribute to this immunosuppressive environment [3]. One strategy to overcome these barriers is through tumor-localized activation of pattern-recognition receptors that are expressed by innate immune cells. Agonists, such as CpG oligodeoxynucleotides (CpG) that bind to Toll Like Receptor 9 (TLR9), have been evaluated as therapeutic adjuvants in cancer vaccine strategies and have shown efficacy in inducing antigen-specific adaptive antitumor immune responses in animal models [4]. However, early-stage clinical trials evaluating direct injection of CpG into gliomas have been less promising [5–7]. It is unclear what factors are responsible for the unsatisfactory response in clinical trials: some possibilities are the sequence of the CpG oligonucleotide tested, rapid degradation of the CpG, or rapid diffusion of the CpG away from the injection site.

Because nanoparticles are rapidly taken up by phagocytic cells, we hypothesized that loading CpG onto nanoparticles would improve the uptake and retention of CpG at the tumor site [8]. Furthermore, because TLR9 recognition of CpG occurs in the endosomal compartment, using a nanoparticle to deliver CpG into the endosome may be an attractive approach for enhancing the efficacy of CpG treatment [9, 10]. In previous work, we demonstrated that single-walled carbon nanotubes (SWCNTs) are efficient, nontoxic carriers of macromolecules into tumor inflammatory cells, both increasing CpG uptake by immune cells and enhancing the efficacy of CpG treatment [11, 12]. This initial formulation of SWCNT/CpG (SWCNT/CpG-1) robustly activated microglia (MG), macrophages (MP), and natural killer (NK) cells. Remarkably, even a single low-dose intracranial (i.c.) injection of SWCNT/CpG-1 (but not free CpG nor the SWCNT control) eradicated GL261 gliomas in 50–60% of mice [11]. In addition, mice that were cured by this treatment developed antitumor immunity and rejected secondary gliomas that were injected into either the brain or into subcutaneous (s.c.) tissue. This therapeutic strategy was also effective in a B16F10 metastatic melanoma model. When administered i.c. (but not s.c.) SWCNT/CpG-1 impaired the growth of both i.c. and s.c. melanomas [12]. These findings suggest that enhanced delivery of immunostimulatory molecules into the brain using nanoparticles as carriers can induce robust local and systemic antitumor immune responses.

Although these initial studies provided the proof of principle that SWCNT/CpG can be successfully used as an immunotherapeutic regimen to treat gliomas, we sought to evaluate an improved SWCNT/CpG formulation (SWCNT/CpG-2) under a more clinically-relevant setting. Following implantation in the mouse brain, the cell line used in our previous studies (GL261) typically forms a spherical tumor with limited invasion into healthy brain. Here we report the treatment of a more invasive and aggressive mouse glioma model (K-Luc) that better recapitulates human disease. The synthesis of SWCNT/CpG was modified (SWCNT/CpG-2) to improve the throughput of material and to include a CpG sequence previously evaluated in humans [5, 6]. In addition, SWCNT/CpG-2 was combined with metronomic doses of temozolomide (TMZ), a cytotoxic chemotherapy agent that is part of the standard of care for glioma patients. TMZ is reported to have immunomodulatory properties so we sought to determine if it also potentiated SWCNT/CpG immunotherapy [13–18]. SWCNT/CpG-2 showed anti-tumor activity in this invasive glioma model and, surprisingly, its efficacy was enhanced when combined with TMZ. This enhanced anti-tumor efficacy was correlated with increased tumor-specific cytotoxic activity in splenocytes. These results reinforce the emerging understanding that immunotherapy can be enhanced by combining it with chemotherapy and support the continued development of SWCNT/CpG therapy.

## Materials and Methods

### Reagents

1,2-distearoyl-*sn*-glycero-3-phosphoethanolamine-N-[amino(polyethylene glycol)-2000] (ammonium salt) (Lipid-PEG-NH_2_) was purchased from Avanti Polar Lipids, Inc. Dithiotheritol (DTT) was purchased from Sigma-Aldrich. Fully phosphorothioated CpG-28 oligonucleotides bearing a terminal dithiol (5’-HO-C_6_-SS-C_6_-TAAACGTTATAACGTTATGACGTCAT-3’) (RSS-CpG) was provided by the DNA core facility at Beckman Research Institute at City of Hope. Sulfosuccinimidyl 6-(3'-[2-pyridyldithio]-propionamido)hexanoate (Sulfo-LC-SPDP) was purchased from ProteoChem (catalogue #c1118). Illustra NAP-25 columns (catalogue #17-0852-01) were purchased from GE Healthcare. Ultra pure SWCNTs (<1% metal impurities) were purchased from NanoIntegris in powder form. 1,2-dioctadecanoyl-sn-glycero-3-phosphoethanolamine (DSPE) was purchased from Avanti Polar Lipids (#850715P). Heterobifunctional discrete PEG (NHS-dPEG_36_-SPDP) was purchased from Quanta Biodesign (#10867). Spin dialysis was performed using 3kD pore-size Amicon Ultra centrifugal filter units (Millipore, UFC900324). All HPLC-grade solvents were purchased from Sigma-Aldrich and Fisher. Agilent autosampler vials (#5183–2072) were used as 2 mL reaction vessels.

HPLC purification and LC-MS analyses were performed on an Agilent 1100 series equipped with a binary pump (Agilent, G1312A), autosampler (Agilent, G1367A), 5 mL manual injector (Wyatt Technology, WISH-01), a diode array detector (DAD) (Agilent, G1315B), and a Quadrupole LC-MS (Agilent, 6120). For prep-scale purification, the mass spectrometer was disconnected and fractions were manually collected after the sample passed through the DAD. Ultrasonication was performed using a QSONICA Sonicator Q700 (QSONICA, Newtown, CT, USA) equipped with a cup horn cooled with running water from the sink.

### Cell culture

Luciferase-expressing KR158B cells (or K-Luc), an invasive glioma cell line that was derived from spontaneous gliomas in *Trp53/Nf1* double-mutant mice in Dr. Tyler Jacks laboratory, was a generous gift from Dr. John Sampson [19]. The GL261 cell line was a generous gift from Dr. Karen Aboody. Both cell lines were cultured in DMEM medium supplemented with 10% FBS (BioWhittaker, Walkersville, MD), 100 U/mL penicillin-G, 100 μg/mL streptomycin and 0.01 M HEPES buffer (Life Technologies, Gaithersburg, MD) in a humidified 5% CO2 atmosphere, and their tumorigenicity was authenticated by histological characterization of intracranial gliomas in mice.

### Tumor implantation and treatments

All animals were housed and handled in accordance to the guidelines of City of Hope Institutional Animal Care and Use Committee (IACUC). The experiments described here were specifically approved by the City of Hope Institutional Animal Care and Use Committee (IACUC) protocol #06030. Intracranial tumor implantation was performed as previously described [20]. Briefly, either K-Luc cells or GL261 cells were harvested by trypsinization, counted, and resuspended in PBS. Female C57BL/6 mice (Jackson Laboratory, Bar Harbor, ME) weighing 15–25 g were anesthetized by intraperitoneal administration of ketamine (132 mg/kg) and xylazine (8.8 mg/kg), and immobilized in a stereotactic head frame. Intracranial (i.c.) tumor implantation was performed by injecting 3 μl of PBS containing 1 ☓ 10^5^ tumor cells through a small burr hole. Tumor cells were injected 2.5 mm right of midline and 1 mm forward of bregma at a depth of 2.5 mm. Four and eight days after i.c. tumor implantation, mice received intratumoral (i.t.) injections of PBS (control, 5 μl), RSS-CpG (5 μg/5 μl water), and SWCNT/CpG-2 (5 μg SWCNT/5 μg CpG/5 μl water). The treatment injections on days 4 and 8 were made at the same coordinates as the tumor implantation (2.5 mm right of midline, 1 mm forward of bregma, 2.5 mm deep). Temozolomide (TMZ, Sigma-Aldrich) treatments were administered i.p. 2.5mg/kg/d/mouse daily, beginning 4 days after tumor implantation. The stock solution (25mM) was prepared by diluting 25 mg of TMZ into 5.15 ml DMSO. For injections, 515 μl of stock solution was added to 9.48 ml of PBS to give a 2.5 mg/10ml injection solution. For the survival experiments, TMZ treatment continued until the animal died. For all other experiments, TMZ treatment was stopped one day before tissue collection.

For the tissue analysis experiments (flow cytometry, IHC, PCR, isolate macrophage from bone marrow and Western blotting), euthanasia (by CO_2_) was carried out within 24 or 48 hours following the final administration of chemical agent or if any sign of illness is observed.

During experiments, animals were monitored daily. Animals displaying excessive pain or discomfort were given appropriate analgesic treatment (Bupernex 0.05–0.1mg/kg SQ/ approximately 0.13–0.2mL) if necessary. For the survival experiments, animals that exhibited mild to moderate signs of distress (hunched, ruffled, decrease in activity, and body weight loss up to 20%) were monitored closely (at least daily) for up to 3 consecutive days to determine if the signs were transient and due to the inflammatory response or chemotherapy treatment. If at 3 days, the mice are still exhibiting one of the mentioned signs, they were euthanized by CO_2_. If at any time animals had a seizure, could not reach food or water (due to impaired ambulation, for example), were unable to remain upright, or lost greater than 25% body weight, they were euthanized immediately.

### SWCNT/CpG-2 synthesis

Lipid-PEG-NH_2_ (21 eq, 12mM) and Sulfo-LC-SPDP (17 eq, 10mM) were dissolved in deionized water and stirred for approximately 2 hours at room temperature. Meanwhile, RSS-CpG (1 eq) was dissolved in deionized water containing 2.5mM DTT and stirred for 2 hours at room temperature. DTT-treated RSS-CpG was then passed through a nap-25 column in order to remove excess DTT. The CpG solution was then added to the Lipid-PEG-NH_2_-containing reaction and heated in a 60°C oil bath for approximately 2 hours. After 2 hours, the reaction was isopropanol-precipitated, dialyzed against deionized water using a 3.5kD MWCO membrane, and lyophilized. A typical reaction was performed with 20 mg of RSS-CpG starting material.

The lyophilized material was dissolved in deionized water (typically at an effective CpG concentration of 1.5 μg/μL) and the concentration measured by UV absorbance (RSS-CpG ε_260nm_ = 261900 L mol^-1^ cm^-1^). SWCNT powder was massed into a tube and the reaction product solution was added until the appropriate SWCNT to CpG mass ratio was reached. The sample tube was ultrasonicated for a process time of 3 h (80 amplitude, 15 s on, 15 s off). After sonication, 6 equivalents of Lipid-PEG-NH_2_ were added to the sample and sonication was repeated for a process time of 3 h–6 h (80 amplitude, 15 s on, 15 s off). The resulting dispersion was then diluted to an effective CpG concentration of 1 μg/μL and 1 μg/μL SWCNTs in deionized water.

### Lipid-PEG-CpG and SWCNT/Lipid-PEG-CpG synthesis

The synthesis of Lipid-PEG-CpG (S2A Fig, MW = 11057.18 g/mol) was based on the procedures reported by Kim *et al*. and Zalipsky [21, 22]. In order to synthesize the intermediate (DSPE-PEG-SPDP, MW = 2602.31 g/mol), 9.8 mg NHS-dPEG_36_-SPDP (5.0 μmol), 5.2 mg DSPE (7.0 μmol), 185 μL Chloroform, and 3 μL triethylamine (TEA) (21.5 μmol) were combined (in that order) in a 2 mL reaction vessel, forming a cloudy solution at room temperature. The reaction was stirred under a positive pressure of N_2_ in a 50°C oil bath for 30 min, then cooled to room temperature and stirred until the reaction went to completion (about 1 hour). The reaction was monitored via LC-MS. The reaction was considered complete when the NHS-PEG-SPDP starting material peak at t = 8.75 min disappeared and was replaced by the DSPE-PEG-SPDP product peak at t = 13 min. (Column: Waters, Acquity UPLC BEH Phenyl 1.7 μm, 2.1 x 50mm, #186002884. Method: Autosampler, flow rate = 0.3 mL/min, column temp = 65°C, A = 0.1% TFA in water, B = 0.1% TFA in acetonitrile, equilibrate the column in 5% B and hold 2 min after injection, 10 min gradient to 100% B, hold 100% B for 4 min. Absorbance was monitored at both 220 nm and 254 nm. MS parameters–positive ion mode, API-ES, nebulizing gas–nitrogen, nebulizer pressure = 35 psig, capillary voltage = 4000 V, drying gas temperature = 350°C, drying gas flow rate = 10.0 L/min, mass range = 110.00–1500.00, 2 min delay before collecting mass spectra). In order to remove excess DSPE starting material, the reaction crude was transferred to a microcentrifuge tube, dried with flowing N_2_, and resuspended in acetonitrile. The solution was stored at 4°C for 1 hour in order to precipitate unreacted DSPE [22] and centrifuged at 10,000xg for 10 min. A portion of the supernatant was transferred to a clean 2 mL reaction vessel and dried under flowing N_2_. The measured yield was 81% (10.5 mg) and 2.5 mg of DSPE-PEG-SPDP was used for the next step of the reaction.

Before beginning the synthesis of DSPE-PEG-SPDP, the RSS-CpG reduction reaction was initiated. In order to reduce RSS-CpG to HS-CpG, 167 μL of a 6 mg/mL solution of RSS-CpG (MW = 8698.25 g/mol, ε_260nm_ = 261900 L·mol^-1^cm^-1^, 0.23 μmol, 2 mg) in deionized water was mixed with an equal volume of freshly-made buffered DTT solution (200mM sodium bicarbonate pH = ~9, 200mM DTT) to yield a 100mM sodium bicarbonate buffer containing 100mM DTT and 3 mg/mL RSS-CpG. Although a stock solution of the sodium bicarbonate buffer was stored for long periods, the DTT was only added to an aliquot of the buffer immediately before the reaction (DTT rapidly oxidizes in solution). The reduction reaction was incubated for 3 h at room temperature. LC-MS analysis was used to confirm that the reduction reaction went to completion. The reduction reaction was considered complete if the elution trace only showed the HS-CpG peak at t = 6.9 min. (Column: Waters, Acquity UPLC BEH Phenyl 1.7 μm, 2.1 x 50mm, #186002884. Method: Autosampler, flow rate – 0.3 mL/min, column temp = 65°C, A = 200mM HFIP/8mM TEA in water, B = 100% methanol, equilibrate the column at 5% B and hold for 2 min after injection, 10 min gradient to 100% B, hold 100% B for 4 min. Absorbance was monitored at both 220 nm and 254 nm. MS parameters–negative ion mode, API-ES, nebulizing gas = nitrogen, nebulizer pressure = 35 psig, capillary voltage = 3500 V, drying gas temperature = 350°C, drying gas flow rate = 10.0 L/min, mass range = 360.00–1500.00, 2 min delay before collecting mass spectra). Spin dialysis was then used to remove the excess DTT and reduction side product. The crude reduction reaction was transferred to a 3kD centrifugal filter and diluted to 15 mL with 200mM acetic acid buffer (pH = 4.5, pH adjusted with ammonium hydroxide). The solution was then centrifuged until <1.5 mL was retained. The sample was then washed 1 more time with acetic acid buffer and two times with 1xPBS. By using absorbance at 260 nm to measure concentration, it was determined that the recovered solution contained 1.67 mg HS-CpG (0.195 μmol, MW = 8566.03 g/mol, ε_260nm_ = 261900 L·mol^-1^cm^-1^) in 1xPBS.

In order to synthesize Lipid-PEG-CpG, a 1.125 mL solution of HS-CpG in 1xPBS (0.174mM, ε_260nm_ = 261900 L·mol^-1^cm^-1^) was added to the reaction vessel containing 2.5 mg of dried DSPE-PEG-SPDP (0.96 μmol, 0.854mM). This reaction was stirred at room temperature overnight and LC-MS was used to verify that the reaction went to completion The reaction was considered complete when the HS-CpG peak at t = 6.9 min disappeared and was replaced by the Lipid-PEG-CpG peak at t = 11.1 min. (Column: Waters, Acquity UPLC BEH C18 1.7 μm, 2.1 x 50 mm column, #186002350, Method: Autosampler, flow rate = 0.3 mL/min, column temp = 65°C, A = 200mM HFIP/8mM TEA in water, B = 100% methanol, equilibrate the column at 5% B and hold for 30 s after injection, 2 min gradient from 5% B to 50% B, 14 min gradient from 50% B to 100% B, hold 100% B for 4 min. Absorbance was monitored at both 220 nm and 254 nm. MS parameters–negative ion mode, API-ES, nebulizing gas = nitrogen, nebulizer pressure = 35 psig, capillary voltage = 3500 V, drying gas temperature = 350°C, drying gas flow rate = 10.0 L/min, mass range = 360.00–1500.00, 2 min delay before collecting mass spectra). The crude reaction was then dried via speed-vac and resuspended in 4 mL of deionized water. In order to isolate pure Lipid-PEG-CpG, the resuspended crude reaction mixture was then HPLC-purified (S2B Fig shows a representative absorbance trace) (Column: Waters, XBridge Prep C18 5μm, 10 x 100mm, #186003255. Method: Autosampler, flow rate = 5.0 mL/min, column temp = 65°C, A = 200mM HFIP/8mM TEA in water, B = 100% methanol, equilibrate the column at 5% B and hold for 30 s after injection, 2 min gradient to 50% B, 14 min gradient to 100% B, hold 100% B for 4 min. Absorbance was monitored at both 220 nm and 254 nm. Fractions were manually collected in 15 mL conical tubes. Fractions were only collected within the peaks. For each run, repeated injections of 200 μL were made until the sample was completely loaded on the column. The product was purified across 4 HPLC runs). The purity of the isolated fractions was verified using the same LC-MS method described above for Lipid-PEG-CpG reaction monitoring. The fractions containing pure product were combined and spin dialysis with deionized water was used to both remove residual salts and methanol. In order to desalt the Lipid-PEG-CpG prior to animal injection (exchange the organic counterions for biocompatible sodium counterions), spin dialysis was used to wash Lipid-PEG-CpG two times with 100mM NaCl and five times with deionized water. The purified Lipid-PEG-CpG was then lyophilized and stored at -20°C. This first batch of Lipid-PEG-CpG was used to treat naïve mice in S2F Fig and was used to construct the SWCNT/Lipid-PEG-CpG in S2G Fig.

A second batch of Lipid-PEG-CpG was prepared and used in the animal experiments shown in S2H–S2I Fig. This batch was prepared using the above protocol with the following changes. The reactions were scaled-up to 13.0 mg DSPE + 24.4 mg NHS-dPEG-SPDP + 463 μL chloroform + 5.2 μL TEA for the DSPE-PEG-SPDP reaction, 10 mg RSS-CpG for the reduction reaction, and 8 mg HS-CpG + half of the DSPE-PEG-SPDP crude for the Lipid-PEG-CpG reaction. After an extended storage of the purified product in solution phase at 4°C, a second HPLC-purification was performed (S2D–S2E Fig) (Column: Waters, XBridge Prep Phenyl 5 μm, 10 x 100mm, #186003272. Method: Manual injector (5 mL), flow rate = 5.0 mL /min, column temp = 45°C, A = 100mM HFIP/4mM TEA in water, B = methanol, equilibrate at 5% B and hold 2 min after injection, 2 min gradient from 5% B to 35% B, 30 min gradient from 35% B to 75% B, hold for 6 min at 75% B, step gradient to 100% B and hold for 5 min. Absorbance was monitored at both 220 nm and 254 nm. Fractions were collected manually into 15 mL conical tubes. Fractions were only collected within the peaks. For each run, 2 manual injections of ~5 mL were performed. Product was purified across 3 HPLC runs). LC-MS analysis of the product from this second HPLC-purification was performed on a phenyl-hexyl column instead of a C18 column. (Column: Phenomenex, Kinetex 2.6u Phenyl-Hexyl 100Å, 50 x 3.0 mm column, #00B-4495-YO. Method: HPLC parameters–Autosampler, flow rate = 0.9 mL/min, column temp = 45°C, A = 100mM HFIP/4mM TEA in water, B = methanol, equilibrate at 5% B and hold 1 min after injection, 0.5 min gradient from 5% B to 40% B, 4.5 min gradient from 40% B to 70% B, hold at 70% B for 2 min, step gradient to 100% B and hold for 2 min. Absorbance was monitored at both 220 nm and 254 nm. MS parameters–negative ion mode, API-ES, nebulizing gas = nitrogen, nebulizer pressure = 55 psig, capillary voltage = 3500 V, drying gas temperature = 350°C, drying gas flow rate = 13.0 L/min, mass range = 360.00–1500.00, 1 min delay before collecting mass spectra).

Prior to SWCNT dispersion or animal injection, Lipid-PEG-CpG was passed through a 0.22 μm sterile filter and tested for endotoxin contamination. For endotoxin testing, a sample aliquot was adjusted to a final concentration of 1 mg/mL in sterile MilliQ water. Endotoxin testing was performed by the Department of Immunology at City of Hope using the Endosafe Portable Test System (Charles River Labs) and a 0.005 EU/mL sensitivity Endosafe—Licensed PTS Endotoxin Cartridge (catalog no. PTS20005F, Charles River Labs) following the manufacturer’s instructions. Briefly, the sealed Endosafe cartridge was removed from the refrigerator and allowed to warm to room temperature. When prompted, the cartridge was inserted into the Endosafe-PTS reader and pre-warmed. The sample was diluted 1:80 with 10mM MgCl_2_ in depyrogenated borosilicate vials and, when prompted, 25 μL of diluted sample was added to each well of the cartridge. In order for the assay to pass the internal suitability test, the spike recovery must be 50%–200% and the coefficient of variation must be <25%. The sample passed the internal suitability test and contained <0.4 EU/mL (based on the 1 mg/mL solution).

To prepare SWCNT/Lipid-PEG-CpG, SWCNTs were dispersed via sonication in a sterile-filtered solution of Lipid-PEG-CpG. The paper-like as-received SWCNTs were torn into smaller pieces with forceps and massed on a thermogravimetric analyzer (TGA Q50, TA Instruments) (±0.1 μg, mass accuracy of ≤0.1%). After massing, the SWCNTs were placed in a 1.5 mL microcentrifuge tube (catalog no. 1615–5500, USA scientific) and autoclaved. After autoclaving, the SWNTs were handled and stored under sterile conditions. To the microcentrifuge tube containing 220 μg SWCNTs was added 86 μL of a 0.293mM solution of Lipid-PEG-CpG in deionized water (0.025 μmol, the molar equivalent of 220 μg RSS-CpG, 0.293mM is the molar equivalent of 2.55 mg/mL RSS-CpG). The sample was sonicated in a cup horn sonicator for a total process time of 60–70 min (6–7 10 min intervals, amplitude = 100, cooled with ice water that was changed between each interval, the sample tube was positioned over the center of the sonicator probe <5 mm from the surface of the probe). After sonication, the sample was diluted to 1 mg/mL SWCNTs with sterile deionized water and stored at 4°C. Using the same protocol, another batch of SWCNT/Lipid-PEG-CpG was made with 102 μg SWCNTs and the same concentrations and ratios of material. These two batches were then combined for the animal experiment. Immediately prior to the animal injection, the SWCNT/Lipid-PEG-CpG was mixed with an equal volume of 2xPBS in order to yield an injection solution of 1 mg/mL SWCNTs, 0.115mM Lipid-PEG-CpG (the molar equivalent of 1 mg/mL RSS-CpG), and 1xPBS.

### RNA extraction and quantitative real-time PCR analysis

PBMCs were isolated by Ficoll density gradient centrifugation of discard peripheral blood samples of healthy donors acquired at the City of Hope Blood Donor Center (http://www.cityofhope.org/blood-donor-center) under City of Hope Institutional Review Board (IRB)-approved protocol (10108). Sample collections were exempt from consenting. Experiments with human materials were performed according to protocols approved by the institutional review committee. The purity of freshly isolated CD14+ monocytes was more than 80% as analyzed by flow cytometry. Monocytes were cultured in 12-well plates with RPMI 1640 medium containing 10% FBS. M-CSF and GM-CSF were purchased from PeproTech and used at a final concentration of 33 ng/ml. Cytokines were added to cultures every 3 days.

PBMCs were incubated with RSS-CpG (1 μg in 1 mL of cell media), SWCNT/CpG-2 (1 μL of 1 mg/mL stock in 1 mL of culture media) or PBS (1 μL in 1 mL media). At various times, cells were collected and total RNA was isolated using the Trizol system (Invitrogen) followed by double DNase treatment and column purification using the Qiagen RNeasy Clean-up Protocol. Real-time PCR was performed in a TaqMan 5700 Sequence Detection System (Applied Biosystems) as described previously [23]. PCR conditions were optimized such that a minimum of 10,000-fold range could be detected for each primer. hGAPDH: 5’-TGCACCACCAACTGCTTAGC-3’ and 3’-GGCATGGACTGTGGTCATGAG-5’. hTNFα: 5’-CCTGCCCCAATCCCTTTATT-3’ and 3’-CCCTAAGCCCCCAATTCTCT-5’. IL-1β: 5’-CCCTAAACAGATGAAGTGCTCCTT-3’ and 3’-GTAGCTGGATGCCGCCAT-5’. hIL-12β: 5’-TGGAGTGCCAGGAGGACAGT-3’ and 3’-TCTTGGGTGGGTCAGGTTTG-5’.

### *In vitro* NF-κB assay

RAW-Blue™ mouse macrophage reporter cells (Invivogen) were cultured to measure TLR-mediated immune stimulation following the manufacturer’s protocol. Briefly, cells (5000 cells in 100 μL in a 96-well dish) were treated with 1 μL of PBS, RSS-CpG (1μg/μl) or SWCNT/CpG-2 (1μg/μl) for 16 hours. RAW-Blue™ cells induce the activation of NF-κB and AP-1, and subsequently the secretion of quantifiable secreted embryonic alkaline phosphatase (SEAP). The level of secreted embryonic alkaline phosphatase (SEAP) was quantified using QuantiBlue™ substrate (InvivoGen) and measured at 620 nm absorption using DTX 880 Multimode Detector (Beckman Coulter). QUANTI-Blue substrate was prepared according to manufacturer’s protocol (InvivoGen).

### Immunofluorescence and 3D reconstruction

Frozen brain sections were prepared from normal and tumor-bearing mice. Immediately after harvest, brains were fixed in paraformaldehyde for four hours before storage in 30% sucrose solution. Brains were embedded in O.C.T. (Tissue-Tek) and 10 μm sections were cut using cryostat (Leica Microsystem Inc., Bannockburn, IL). Prior to immunofluorescence staining, slides were baked at 37°C and permeabilized in methanol for 15 minutes. Slides were incubated with GFAP (1:100, rat anti-GFAP, Life Technologies, Carlsbad, CA), CD11b (1:20, rat anti-mouse, Abcam, Middlesex, NJ), CD8 (1:20, rat anti-mouse, Abcam) or CD3 (1:20, rat anti-mouse, R&D Systems, Minneapolis, MN) primary antibodies for 2 hours. Slides were washed with PBS three times for 5 minutes and incubated with secondary antibody (Goat anti-rat Alexa Fluor 555, 1:200 dilution, Life Technologies) for another hour. Tissue sections were mounted in Vectashield mounting medium containing 4060-diamidino-2-phenylindole (DAPI) (Vector, Burlingame, CA), imaged with AX-70 fluorescent microscopy (Leica Microsystems Inc., Bannockburn, IL) and prepared by Zeiss LSM Image Browser software. In immunofluorescence images, the black SWCNTs were false colored white by inverting the colors of the phase image and adjusting the contrast so that the background was entirely black. For the 3D imaging, slides were digitized using the NanoZoomer HT Scan System capable of scanning whole slides (Hamamatsu Photonics, Japan). The digitized whole slides were reconstructed in 3D using Voloom software (MicroDimensions, Germany).

### Flow cytometry analysis

Tumors were harvested and examined by flow cytometry as previously described [11]. Cell suspensions from brain and spleen tissues were forced through a 40 μm filter. Blood samples were lysed. Freshly-prepared samples were resuspended in 0.1M PBS containing 1% FBS and 2mM EDTA and incubated with FcγIII/IIR-specific Ab to block nonspecific binding. Samples were then stained with different combinations of antibodies (CD11b, NK1.1, CD4, CD3, and CD20) or isotype controls for 1 hour at 4°C. For intracellular staining, cells were fixed and permeabilized immediately after cell surface staining and stained with FoxP3 antibody according to the manufacturer's description (Affymetrix eBioscience; Treg staining kit). Inflammatory cells were gated and separated from the remainder of the sorted cells based on forward vs. side-scatter analysis and staining characteristics. The proportion of each cell type was measured as percent of total inflammatory cells. Tumor MPs were gated as CD45^high^ CD11b^+^ and MG as CD45^low^ CD11b^+^, based on a previously described phenotypic characterization [24]. For the detection of apoptosis, Raw-Blue cells were cultured and treated with PBS, RSS-CpG (10 μg/mL), SWCNT/CpG-2 (10 μg/mL). Twenty-four hours after, the cells were stained with Annexin V and propodium iodide (PI) according to the manufacturer's protocol (Apoptosis detection kit; Affymetrix eBioscience). Fluorescence data were collected on a CyAn (BDIS, San Jose, CA) or Fortessa (BD Biosciences). The data were analyzed using FlowJo software (Tree star Inc.).

### Chromium release cytotoxicity assay

Cytotoxicity against K-Luc glioma cells was determined using a standard ^51^Cr release assay [25]. Briefly, effector cells were derived from spleens of K-Luc-bearing C57BL6 mice (*n* = 4) injected i.c. with SWCNT/CpG-2. Mice were sacrificed 48 hours post-treatment and splenocytes were harvested and co-incubated with irradiated (30,000 rad) K-Luc cells for 7 days [26]. Effectors were then co-incubated for 4 hours with 5,000 ^51^Cr-loaded K-Luc targets in 96-well plates at ratios of 100:1, 20:1, and 4:1 (nine replicates). Radioactivity released into the supernatant was measured using a Cobra Quantum gamma counter (PerkinElmer). Percent specific lysis was calculated as: (experimental release—spontaneous release)/(maximum release—spontaneous release) ☓ 100%.

### High-Resolution Mass Spectrometry

Using 3kD MWCO centrifugal spin filters (Amicon Ultra 0.5 mL, catalogue # UFC500324, EMD Millipore), product from the Lipid-PEG-CpG synthesis was washed with 200mM ammonium acetate and deionized water for desalting. The sample was introduced by nanoelectrospray into a Thermo LTQ-FT operated in negative ion mode under manual control. Ions were detected in the ICR cell at resolution 100,000 (at m/z 400).

### Statistical analysis

Statistical comparison was performed with the prism software using one-way analysis of variance (ANOVA) or the Student’s t-test. Survival was plotted using a Kaplan-Meir survival curve and statistical significance was determined by the Log-rank (Mantel-Cox) test. A P value of less than 0.05 was considered significant.

## Results

### Characterization of SWCNT/CpG-2, Lipid-PEG-CpG, and SWCNT/Lipid-PEG-CpG

**Fig 1:**
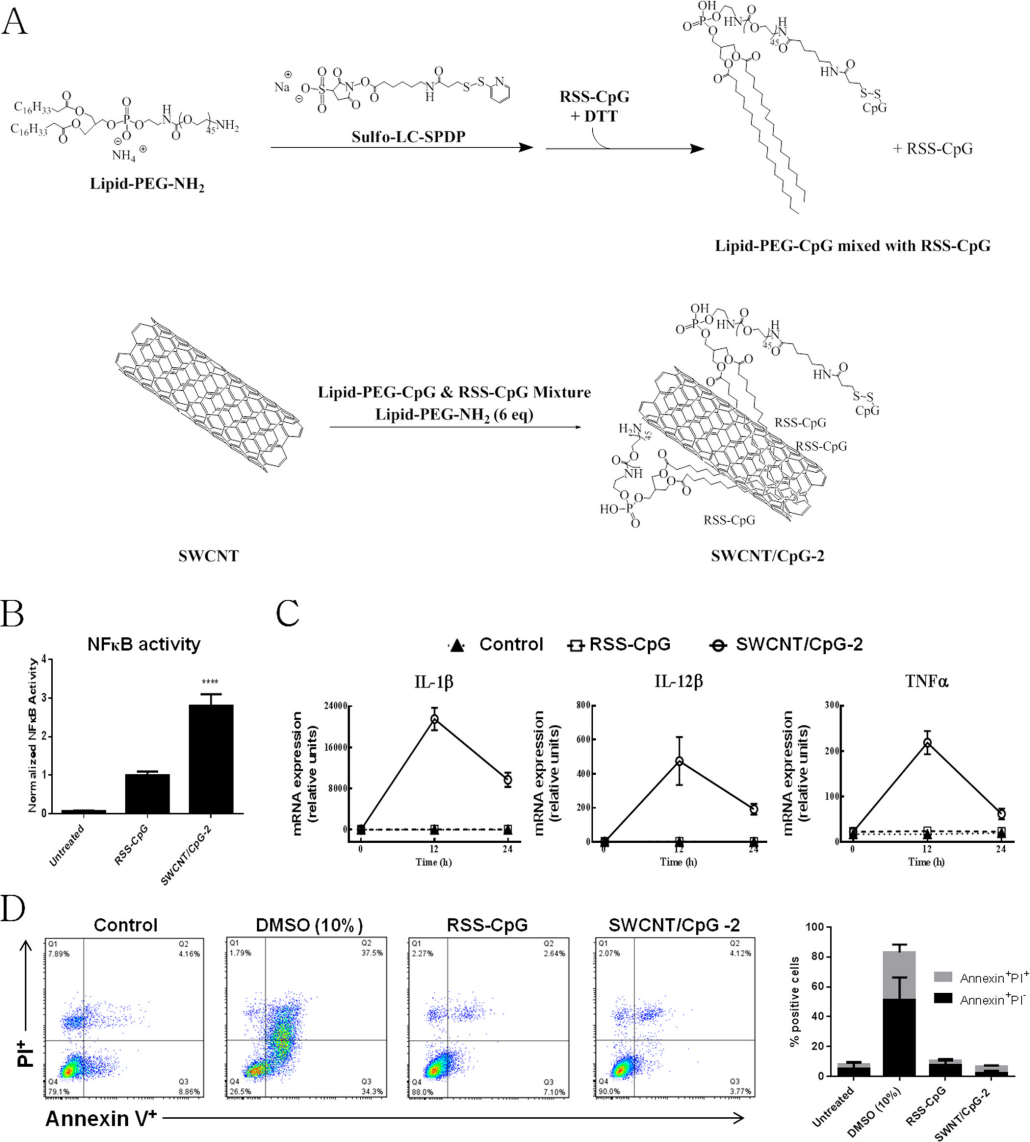
Synthesis and *in vitro* activity of the SWCNT/CpG-2. (A) Schematic drawing of the SWCNT/CpG-2 synthesis. RSS-CpG was reacted with Lipid-PEG-NH_2_ prior to the dispersion of SWCNTs. (B) NFκB activity of RSS-CpG (10 μg/mL), and SWCNT/CpG-2 (10 μg/mL) compared to untreated RAW-Blue™ macrophages. SWCNT/CpG-2 treatment induces a significant increase in NFκB activity when compared to RSS-CpG treatment (Student’s t-test, **** P < 0.0001, n = 6). (C) Real-Time PCR analysis of pro-inflammatory cytokines in human peripheral blood mononuclear cells (PBMCs) incubated with RSS-CpG (1 μg/mL) or SWCNT/CpG-2 (1 μg/mL). (D) Cell toxicity was measured by the Annexin V/PI apoptosis assay. This assay detects cell populations that are indicative of cytotoxicity: apoptotic cells (Annexin^+^ PI^-^, bottom right quadrant of flow plot) and necrotic cells (Annexin^+^ PI^+^, top right quadrant). Raw-Blue™ cells were incubated with SWCNT/CpG-2 (10 μg/mL), RSS-CpG (10 μg/mL) or PBS for 12 hours prior to Annexin V and PI labeling. As a negative control, cells were treated with 10% DMSO in order to induce cell death. Representative flow cytometry analysis (left panel), and a stacked bar chart showing the proportion of both apoptotic and secondary necrotic cells (right panel) (n = 3).

A 2^nd^ generation SWCNT/CpG (SWCNT/CpG-2) synthesis scheme was developed in order to make several improvements on the previous SWCNT/CpG (SWCNT/CpG-1) synthesis–to improve the scalability of material production, to enable characterization of molecules used to coat the nanotubes, and to utilize a CpG sequence (CpG-28) previously tested in humans (Fig 1A) [5, 6]. This synthesis scheme was intended to produce Lipid-PEG-CpG, however, due to an inefficient reduction of RSS-CpG early in the synthesis, the majority of the isolated material at the end of the reaction was RSS-CpG starting material and not Lipid-PEG-CpG (S1A Fig). We simultaneously tested SWCNTs dispersed with this heterogenous mixture composed primarily of RSS-CpG and Lipid-PEG-NH_2_ (collectively referred to as SWCNT/CpG-2) and revised our synthesis to produce SWCNTs coated in pure Lipid-PEG-CpG (SWCNT/Lipid-PEG-CpG) (S2A Fig).

Surprisingly, the heterogenous mixture SWCNT/CpG-2 induced a greater inflammatory response *in vitro* than free RSS-CpG. SWCNT/CpG-2 treatment increased both the NFκB activity in a mouse reporter cell line (Fig 1B) and the expression of pro-inflammatory cytokines IL-1β, IL-12β, and TNFα in human PBMCs (Fig 1C) when compared to treatment with an equivalent amount of free RSS-CpG. No cytotoxicity was observed *in vitro* at the concentrations used (Fig 1D). Because of these promising *in vitro* results, SWCNT/CpG-2 was subjected to further testing in animals.

In an equally surprising turn of events, SWCNTs coated in pure Lipid-PEG-CpG (SWCNT/Lipid-PEG-CpG) showed poor activity. Because recent literature suggested that free Lipid-PEG-CpG may possess enhanced immunostimulatory properties *in vivo*, naïve mice were injected with a range of Lipid-PEG-CpG doses and evaluated for signs of gross toxicity [27]. Lipid-PEG-CpG was found to be non-toxic at all doses tested (up to the molar equivalent of 5 μg RSS-CpG) (S2F Fig). The immunotherapeutic efficacy of both Lipid-PEG-CpG alone and SWCNT/Lipid-PEG-CpG were tested *in vivo*. For preliminary screening *in vivo*, the GL261 glioma model was used because we have previously shown that treatment with a carbon nanotube-CpG construct yielded very clear and dramatic survival benefit [11, 12]. Even when injected at doses that cured mice in our previous studies, neither Lipid-PEG-CpG alone nor SWCNT/Lipid-PEG-CpG showed any increased efficacy when compared to saline injected controls or CpG alone (S2G–S2I Fig). These results suggest that the activity seen with SWCNT/CpG-2 is the result of other components in the mixture and efforts are underway to more fully characterize the impact of each component. For the work presented here, we chose to continue working with SWCNT/CpG-2 based on its intriguing *in vitro* activity.

### Antitumor activity of SWCNT/CpG-2 in animals with invasive gliomas

**Fig 2:**
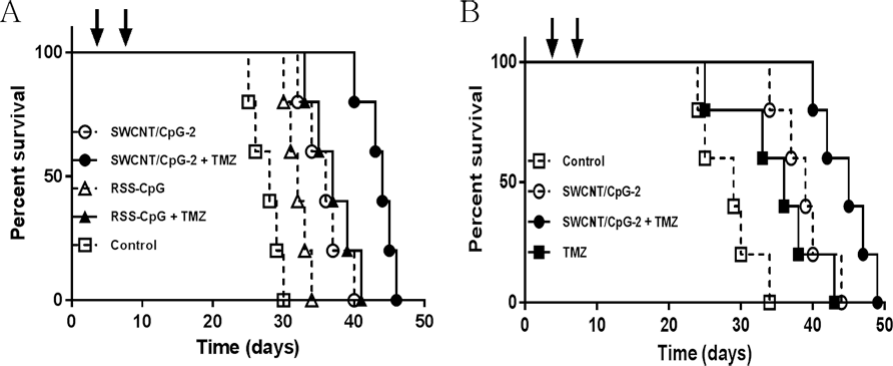
Anti-tumoral efficacy of SWCNT/CpG-2 in the invasive K-Luc glioma model. (A and B) Four and eight days (indicated by black arrows) after i.c. tumor implantation, mice received intratumoral (i.t.) injections of SWCNT/CpG-2, RSS-CpG or PBS. TMZ (2.5 mg/kg) was administered daily beginning on day 4. Kaplan–Meier analysis showed SWCNT/CpG-2+TMZ significantly extended survival relative to all other treatment groups (curves significantly different P < 0.0001 by Log-rank test, n = 5).

To evaluate the antitumor activity of SWCNT/CpG-2 in a more invasive glioma model, mice bearing established i.c. K-Luc gliomas were injected intratumorally with either RSS-CpG or SWCNT/CpG-2 (at equivalent doses of CpG), alone or in combination with subsequent TMZ treatment (Fig 2A and 2B). For these studies, two intratumoral injections of RSS-CpG or SWCNT/CpG-2 were used as this resulted in increased median survival relative to a single injection (S3A Fig). Treatment injections began on day 4. At this time point, the K-Luc tumor is well-established and has formed a substantial tumor that is readily visible by histology (S3C–S3D Fig, a 3D reconstruction of tumor volume and SWCNT distribution 14 days after treatments also included as S3B Fig). It was found that combination treatment with SWCNT/CpG-2 and metronomic doses of TMZ was significantly more effective than the other treatment regimens (Fig 2A and 2B, curves significantly different P < 0.0001 by Log-rank test). In fact, the addition of TMZ significantly enhanced the efficacy of both RSS-CpG and SWCNT/CpG-2 treatment (Fig 2A, P < 0.025 Log-rank test). Unlike our previous observations in the GL261 glioma model [11], animals bearing the more invasive intracranial K-Luc gliomas were not cured after SWCNT/CpG-2 therapy, alone or in combination with TMZ.

### SWCNT/CpG-2-mediated inflammatory response

**Fig 3:**
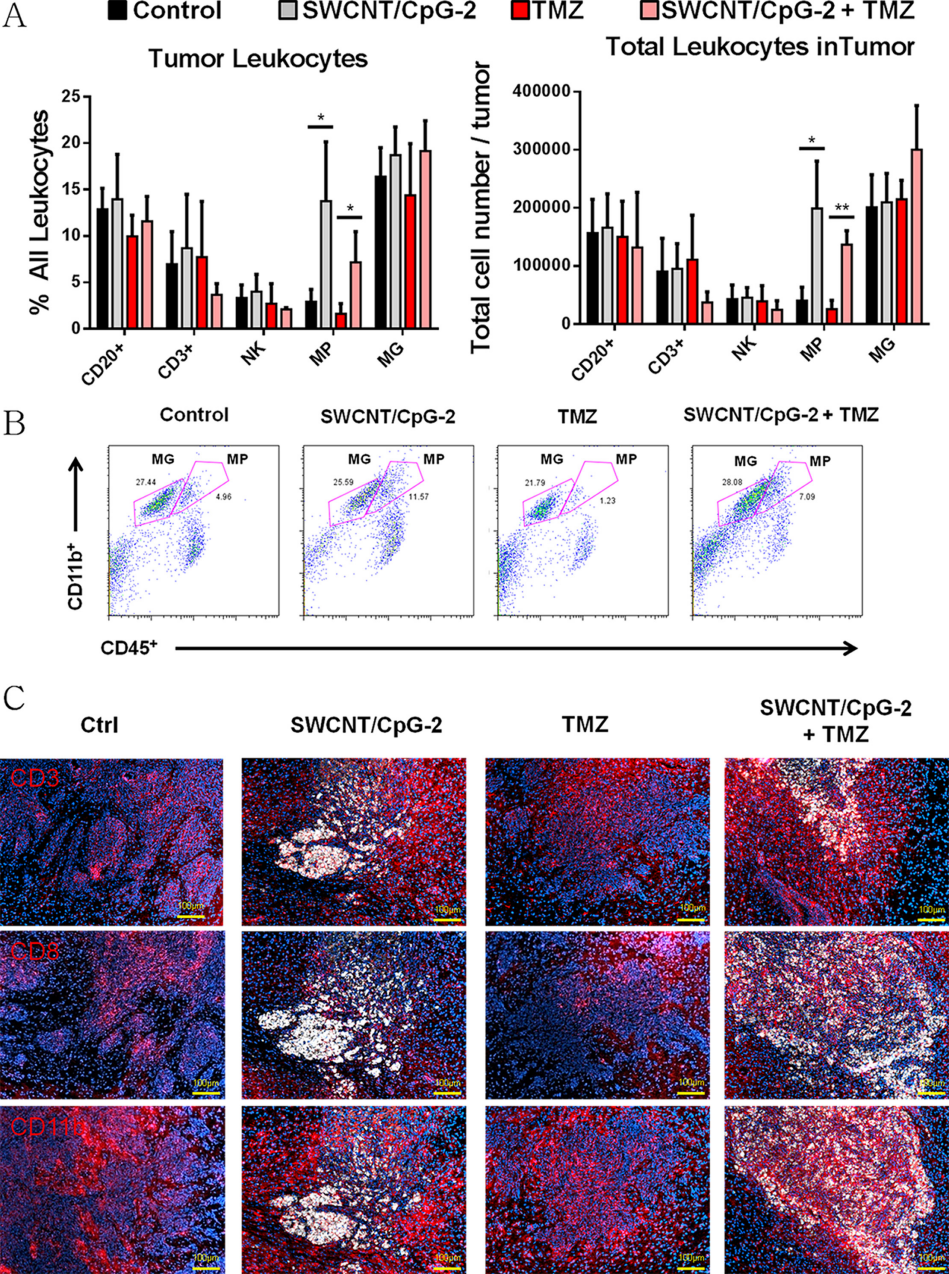
SWCNT/CpG-2 + TMZ treatment increases the proportion of macrophages in the tumor. (A) Proportion (left panel) and absolute number (right panel) of tumor infiltrating CD20^+^, CD3^+^, NK, MP (CD11b^+^CD45^high^) and MG (CD11b^+^CD45^low^) cells (unpaired two-tailed t-test * P < 0.05 and ** P < 0.01, n = 3). (B) Representative flow cytometry data of percent MP (CD11b^+^CD45^high^) and MG (CD11b^+^CD45^low^) cells in tumor after treatments. (C) Representative fluorescent imaging of tumor infiltrating CD3^+^, CD8^+^, and CD11b^+^ cells 10 days after treatment (14 days after tumor implantation). Scale bar is 100 μm.

To study potential mechanisms by which TMZ enhanced SWCNT/CpG-2 anti-tumor efficacy, we next evaluated the local and systemic inflammatory responses. Treatment with SWCNT/CpG-2 resulted in a significant macrophage(MP)-specific infiltration into K-Luc tumors (Fig 3A and 3B). Interestingly, this MP-specific tumor infiltration was also maintained with the addition of TMZ treatment. The overall frequency and distribution of other tumor-associated inflammatory cells like B cells (CD20^+^), T cells (CD3^+^), natural killer cells (NK) and microglia (MG) remained unchanged following treatment with SWCNT/CpG-2, TMZ or the combination (Fig 3A–3C).

### Cytotoxicity of splenocytes following treatment

**Fig 4:**
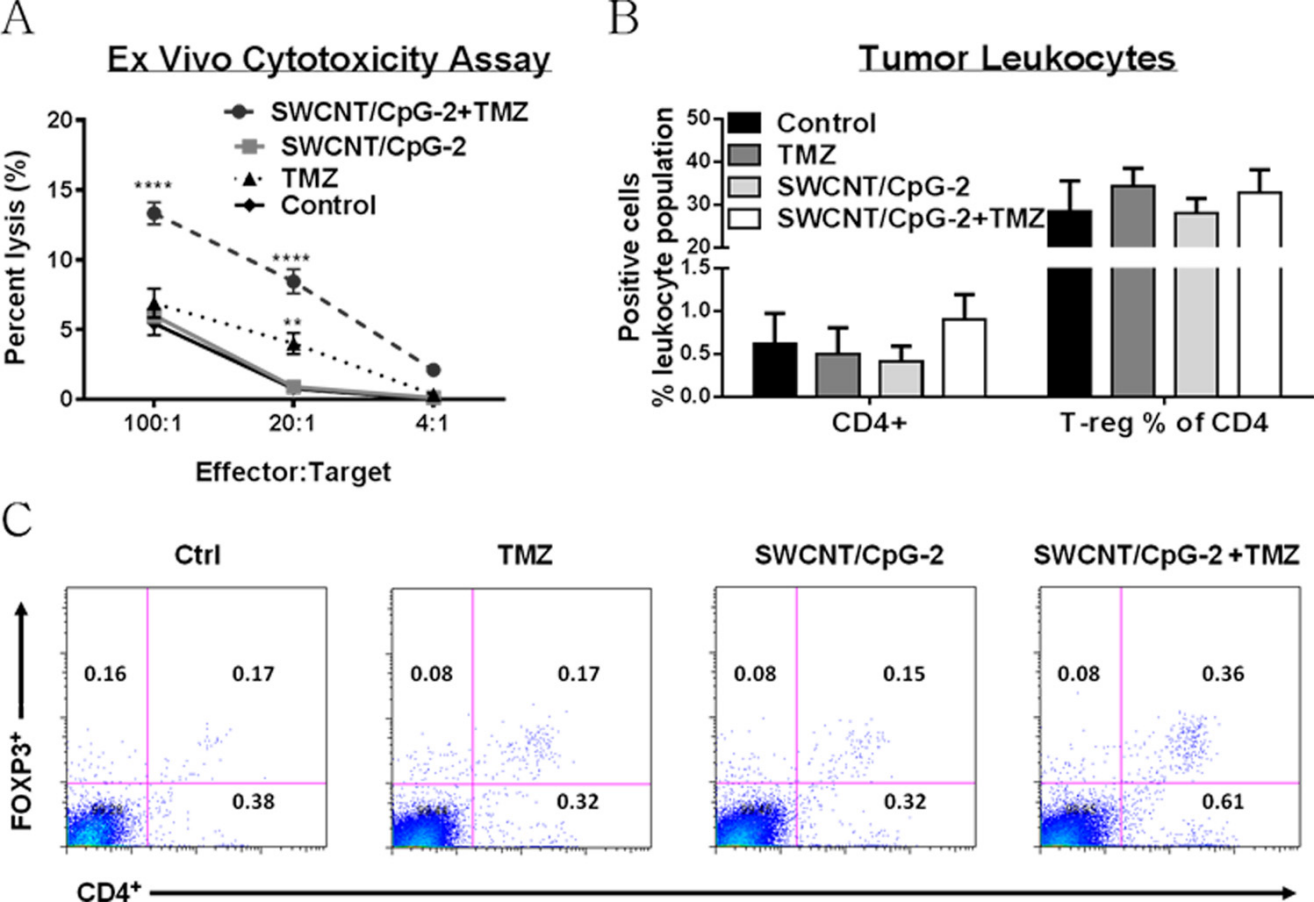
Combination SWCNT/CpG-2 + TMZ enhanced the *ex vivo* cytotoxicity against K-Luc cells. (A) Splenocytes from K-Luc bearing animals were used in a chromium release assay against K-Luc target cells one week after treatment. ^51^Cr-Labled K-Luc tumor cells were used as target cells. Cytotoxicity against K-Luc cells was significantly higher in SWCNT/CpG-2 + TMZ treated mice (one-way ANOVA was performed for each effector:target cell ratio. ** P < 0.01, **** P < 0.0001, n = 9). (B) Proportion of intratumoral CD4^+^ T lymphocytes and regulatory T cells (CD4^+^FoxP3^+^) was measured 10 days after treatments. There was no statistically significant change in either cell population (one-way ANOVA, P > 0.05, n = 3 for TMZ group, n = 4 for other groups). (C) Representative flow cytometry data.

To determine if the enhanced efficacy of SWCNT/CpG-2 and TMZ was associated with increased tumor-killing by effector cells, we performed a chromium release assay on splenocytes isolated from treated animals. The assay showed significantly enhanced tumor killing by splenocytes from the SWCNT/CpG-2 + TMZ treatment group at all effector:target ratios (Fig 4A). In an effort to understand this phenomenon, we evaluated the frequency of Tregs and CD4^+^ cells in tumors after treatment. Low-dose TMZ has been reported to deplete Tregs [18]. Because Tregs contribute to the suppression of immune responses, a reduction in Tregs could explain the increased *ex vivo* tumor cytotoxicity. However, the proportion of Tregs within the tumor-associated CD4^+^ population did not change with TMZ treatment (Fig 4B). The frequency of Tregs and CD4^+^ cells in the spleen and blood were also evaluated and no changes were observed (S4 Fig). Overall, this indicates that treatment with SWCNT/CpG-2 + TMZ increased antitumor cytotoxic activity by a mechanism which does not involve the depletion of tumor-associated Tregs.

### Evaluation of potential toxicity of SWCNT/CpG-2 treatment

**Fig 5:**
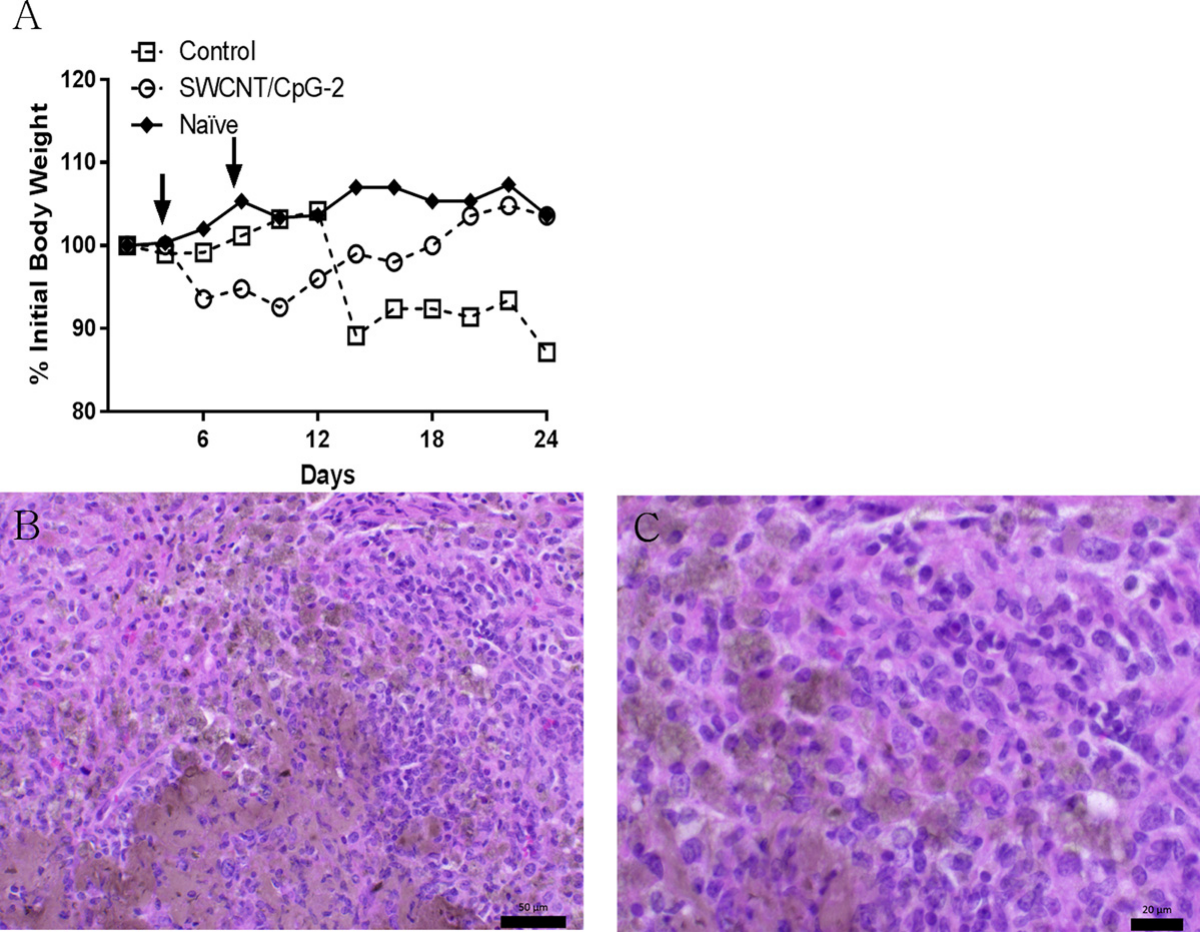
SWCNT/CpG-2 treatment is well tolerated. (A) Mice receiving SWCNT/CpG-2 briefly lost weight before returning to normal weight in the same time frame that control mice became sick and lost weight. Arrows indicate dates of SWCNT/CpG-2 treatment (n = 3 for naïve, n = 5 for control and SWNT/CpG-2). (B and C) Representative H&E-stained brain tissue sections from tumor-bearing mice treated with SWCNT/CpG-2 14 days after tumor implantation. In tissue regions with high SWCNT retention (brown discoloration), there are no signs of cellular damage or necrosis. Scale bar equals (B) 50 μm and (C) 20 μm.

In our studies with tumor-bearing mice, no gross toxicity was observed when mice were treated with SWCNT/CpG. During the study shown in Fig 2B, the body weight of all animals was measured every two days (S1 Table). SWCNT/CpG-2 treated mice experienced a brief but transient weight loss following the treatment. Tumor-bearing control mice, however, experienced dramatic weight loss beginning at day 12 (Fig 5A). Furthermore, pathological analysis of brain tissue from SWCNT/CpG-2-treated mice also showed no signs of toxicity. In tissue containing a high concentration of SWCNTs (brown discoloration in the H&E), the surrounding cells lacked any signs of nuclear changes that would be indicative of cellular injury related to the presence of the nanoparticles (Fig 5B and 5C).

## Discussion

The SWCNT/CpG used in our previous reports (SWCNT/CpG-1) was synthesized by first coating the SWCNTs in Lipid-PEG-NH_2_, followed by ultracentrifugation to remove uncoated SWCNTs, then coupling a linker to the terminal amine of the Lipid-PEG-NH_2_ coating and finally conjugating the CpG through a disulfide bond. Theoretically, this reaction scheme yielded SWCNTs coated with Lipid-PEG-CpG as the desired product. The SWCNT/CpG-1 synthesis protocol had two major drawbacks: 1) low material yield, and 2) inability to characterize the molecules coating the nanotubes. We were unable to characterize the coating on the nanotubes in SWCNT/CpG-1 because the coupling chemistry was all conducted on the nanotubes which interfere with standard characterization techniques. In an effort to solve these two problems, we developed a new synthetic strategy wherein we aimed to prepare Lipid-PEG-CpG in solution (which enabled its characterization), coat it onto SWCNTs (SWCNT/Lipid-PEG-CpG) and use the dispersed material without ultracentrifugation (which improved material yield). In our attempt to carry out this improved synthesis, we made a surprising discovery: although SWCNT/Lipid-PEG-CpG was thought to be the active component of SWCNT/CpG-1, it showed no activity *in vivo*. A revised synthesis protocol produced a second-generation SWCNT/CpG construct with activity *in vivo* (SWCNT/CpG-2). Unlike SWCNT/Lipid-PEG-CpG, SWCNT/CpG-2 is a heterogeneous material, composed of SWCNTs dispersed in a mixture primarily containing RSS-CpG and Lipid-PEG-NH_2_. This suggests that the synthetic requirements for an efficacious SWCNT/CpG construct are quite different and potentially more complex than originally thought. Future experiments will be necessary to determine the optimal ratios of the various components of SWCNT/CpG-2 in order to maximize efficacy.

It was found that the *in vivo* activity of SWCNT/CpG-2 was enhanced by combining it with metronomic doses of TMZ. The exact mechanism by which TMZ enhanced SWCNT/CpG-2 responses remains unclear. The improved survival of mice treated with the combination therapy is likely due to an additive effect. One possible explanation is that TMZ enhanced the antitumor efficacy of SWCNT/CpG-2 through an immune-mediated mechanism. The immunomodulatory effects of some chemotherapeutic agents have been well documented [28, 29]. They include the induction of immunogenic tumor cell death, the promotion of NK or T cell function, and the impairment of immunosuppressive cells (myeloid-derived suppressor and regulatory T cells) by reducing their frequency/number and/or altering their function. Previous studies have shown that low doses of TMZ enhanced antitumor immunity through Treg elimination leading to a reduction in the proportion of Treg cells in the total CD4^+^ population. We have shown that the combination of TMZ with SWCNT/CpG-2 enhanced effector immune cell function. Although the proportion of Treg cells in the total CD4^+^ population remained unchanged, TMZ enhancement of antitumor immunity may have resulted from an alteration in Treg function. Interestingly, our results also demonstrate that SWCNT/CpG-2 treatment induced increased MP infiltration into the tumor in both the presence and absence of concurrent TMZ treatment. The enhanced anti-tumor immunity induced by the combination therapy may have been due to the elimination of suppressive myeloid cells by TMZ and replacement with highly reactive pro-inflammatory effector myeloid cells.

Consistent with our previous reports [11, 12], there was no evidence of gross toxicity following i.c. SWCNT/CpG therapy. When the weights of SWCNT/CpG-2 treated animals were compared to untreated tumor-bearing (Control) and naïve animals, it appeared that SWCNT/CpG-2 treatment caused a transient inflammatory response leading to a brief weight loss. However, SWCNT/CpG-2-treated mice, regained their weight (most likely as a result of temporary tumor regression) while untreated mice rapidly deteriorated. Furthermore, analysis of brain tissue sections from treated mice showed no signs of necrosis or cellular toxicity in the presence of the nanotubes.

Despite general concern in the scientific community about the toxicity carbon nanotubes, our finding that SWCNT/CpG is non toxic is consistent with the literature. An early report identified that MWCNTs could cause asbestos-like symptoms when administered into the abdominal cavity of mice [30]. This heightened the concern around the potential toxicity of carbon nanotubes in general. However, that same publication and many others have shown that SWCNTs are significantly less toxic than MWCNTs [31]. While early work on the toxicology of carbon nanomaterials reached contradictory conclusions, more recent work identified that residual metal contaminants were one of the most significant contributors to the toxicity observed [32, 33]. With this in mind, it is no surprise that purified SWCNTs appear to have minimal toxicity when administered intravenously [34–37]. Even when extremely high doses of SWCNTs were given intraperitoneally or orally, the mice remain healthy over an extended period of time [38]. Remarkably, even when well-dispersed SWCNTs were administered by inhalation to mice, no evidence of pulmonary inflammation was observed [39]. Thus, it has been repeatedly shown that appropriately prepared and purified SWCNTs can be used without significant toxicity at the organ or animal level.

## Conclusion

Here we show that the formulation of efficacious SWCNT/CpG is more complex than previously thought. Preparation of pure SWCNT/Lipid-PEG-CpG, the theoretical product of our previous synthesis, showed no activity *in vivo*. Instead, the active SWCNT/CpG construct is SWCNT/CpG-2, a material composed of SWCNTs dispersed in a heterogenous mixture primarily composed of RSS-CpG and Lipid-PEG-NH_2_. This material has activity in a highly invasive mouse model of glioma. No toxicity was observed following the intratumoral (i.t.) injection of SWCNT/CpG-2. The efficacy of SWCNT/CpG-2 treatment was enhanced by combining it with metronomic doses of temozolomide (TMZ). In contrast with previous studies, the TMZ treatment did not reduce tumor associated Tregs. Rather, combination treatment with TMZ and SWCNT/CpG-2 resulted in effector cells that more efficiently killed their target cell population. Furthermore, combination treatment resulted in an increase in tumor-associated macrophages, yet infiltration of these cells was not as robust when compared to the SWCNT/CpG-2 only treatment group. Although the polarization of myeloid-derived cells was not examined here, these findings suggest that the enhanced anti-tumor immunity with the combination therapy may have been due to the elimination of suppressive myeloid cells and replacement with highly reactive, pro-inflammatory effector myeloid cells. Future studies will investigate this possibility in more detail and will also investigate the composition of SWCNT/CpG-2 in order to define the optimal ratio of components in this heterogenous material.

## Supporting Information

S1 Fig Characterization of SWCNT/CpG-2. (A) Negative ion high-resolution MS of the product from the Lipid-PEG-CpG synthesis reaction. The molecular weight (MW) of the Lipid-PEG-CpG product from this reaction is 11,556 while starting material RSS-CpG is MW = 8698.25 (exact mass = 8691.89) and reduced starting material (HS-CpG) is MW = 8566.03 (exact mass = 8559.83). (B) NFκB activity of SWCNT/CpG-2 conjugates at various SWCNT: CpG ratios. Raw-Blue cells were incubated with each mixture for 12 hours. Values were normalized to the activity of free RSS-CpG (10 μg/mL). (C) Stability of SWCNT/CpG-2 activity after storage at 4°C. NFκB activity was normalized to the activity of cells treated with freshly thawed RSS-CpG (10 μg/mL).
(TIF)

S2 Fig Synthesis and characterization of SWCNT/Lipid-PEG-CpG. (A) Reaction scheme for the synthesis of Lipid-PEG-CpG. The PEG spacer used in this synthesis is discrete instead of the polydispersed PEG-2000 linker used in the original synthetic scheme. Therefore, the mass of the new Lipid-PEG-CpG in panel C is different than the theoretical mass in S1A Fig. (B) Absorbance trace at 254 nm (A254) of the prep HPLC purification (C18 column) of the first batch of Lipid-PEG-CpG (left panel) and the A254 trace (C18 column) of the combined purified fractions (right panel). (C) High-resolution mass spectrum of the Lipid-PEG-CpG isolated in B. (D) A254 trace of the second prep HPLC purification (phenyl column) of the second batch of Lipid-PEG-CpG (left panel) and the A254 trace (phenyl-hexyl column) of a representative purified fraction (right panel). (E) Low resolution mass spectrum from the LC-MS analysis of the peak in D. (F) Lipid-PEG-CpG is non-toxic. Naïve mice treated with various doses of Lipid-PEG-CpG (5 μL of the molar equivalent of 0.001 μg/μL to 1 μg/μL RSS-CpG) showed no signs of gross toxicity (n = 4). (G) GL261-tumor-bearing mice treated with SWCNT/Lipid-PEG-CpG showed no survival benefit compared to control (n = 4). (H) GL261-tumor-bearing mice treated with Lipid-PEG-CpG showed no survival benefit compared to RSS-CpG alone (the concentration of both samples was normalized to the molar equivalent of 1 μg/μL RSS-CpG) (n = 6). (I) GL261-tumor-bearing mice treated with various doses of Lipid-PEG-CpG (5 μL of the molar equivalent of 0.001 μg/μL to 1 μg/μL RSS-CpG) showed no survival benefit compared to control (n = 3).
(TIF)

S3 Fig Histological features of highly invasive K-Luc glioma cells with and without treatment with SWCNT/CpG-2. (A) Survival curve of tumor-bearing mice treated with a single injection (arrow) of either PBS, RSS-CpG, or SWCNT/CpG. Kaplan-Meier analysis showed that a single SWCNT/CpG treatment significantly extended survival when compared to both control and RSS-CpG (Log-rank test, P < 0.05, n = 5), but resulted in a lower median survival when compared to two treatments (34 days in S3A Fig vs 36 days and 39 days in Fig 2A and 2B). (B) 3D image of SWCNT/CpG and K-Luc tumor cells. K-Luc tumor cells (Blue) and SWCNT/CpG (Yellow). (C) H&E staining shows detectable tumor on Day 4 after K-Luc tumor implantation. (D) SWCNT/CpG treatment injected on Day 4 co-localizes with the tumor. The arrows indicate visible SWCNT accumulation (brown discoloration).
(TIF)

S4 Fig Proportion of systemic CD4^+^ T lymphocytes and regulatory T cells following SWCNT/CpG-2 + TMZ treatment. Frequency of CD4^+^ T cells and regulatory T cells (CD4^+^FoxP3^+^) in the spleen (left panel) and blood (right panel) was measured 10 days after treatment (14 days after tumor implantation) (n = 3 for TMZ group, n = 4 for other groups).
(TIF)

S1 Table SWCNT/CpG-2 treatment is well tolerated. Body weight (grams) of mice during the survival experiment in Fig 2B.
(XLSX)
