# Supplementary figures and images for: Metronomic Doses of Temozolomide Enhance the Efficacy of Carbon Nanotube CpG Immunotherapy in an Invasive Glioma Model
Source: PLoS One. 2016 Feb 1;11(2):e0148139. doi: 10.1371/journal.pone.0148139 (PMC4734656; doi:10.1371/journal.pone.0148139)

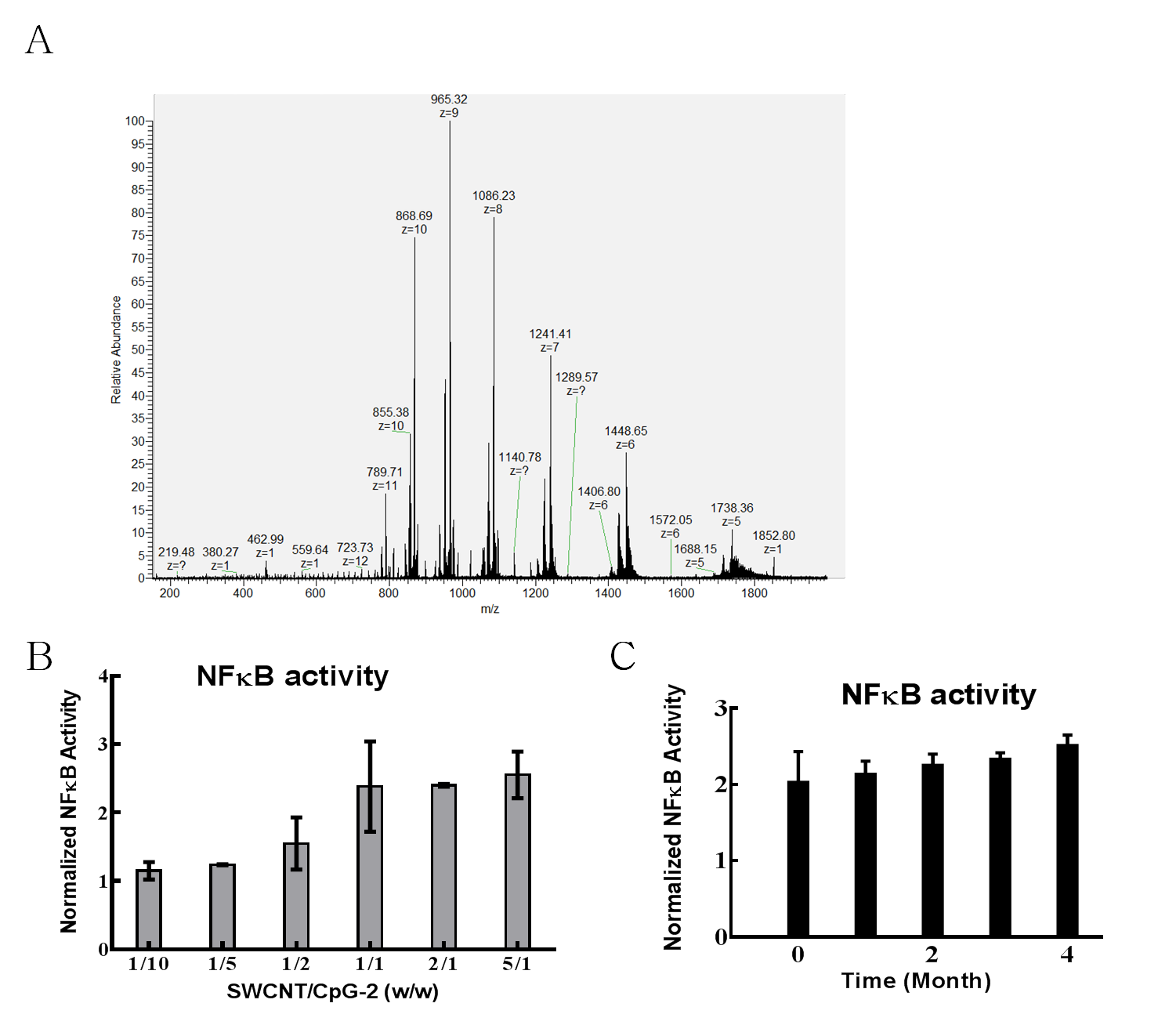

Supplement: S1 Fig — (A) Negative ion high-resolution MS of the product from the Lipid-PEG-CpG synthesis reaction. The molecular weight (MW) of the Lipid-PEG-CpG product from this reaction is 11,556 while starting material RSS-CpG is MW = 8698.25 (exact mass = 8691.89) and reduced starting material (HS-CpG) is MW = 8566.03 (exact mass = 8559.83). (B) NFκB activity of SWCNT/CpG-2 conjugates at various SWCNT: CpG ratios. Raw-Blue cells were incubated with each mixture for 12 hours. Values were normalized to the activity of free RSS-CpG (10 μg/mL). (C) Stability of SWCNT/CpG-2 activity after storage at 4°C. NFκB activity was normalized to the activity of cells treated with freshly thawed RSS-CpG (10 μg/mL). (TIF) [file pone.0148139.s001.tif]

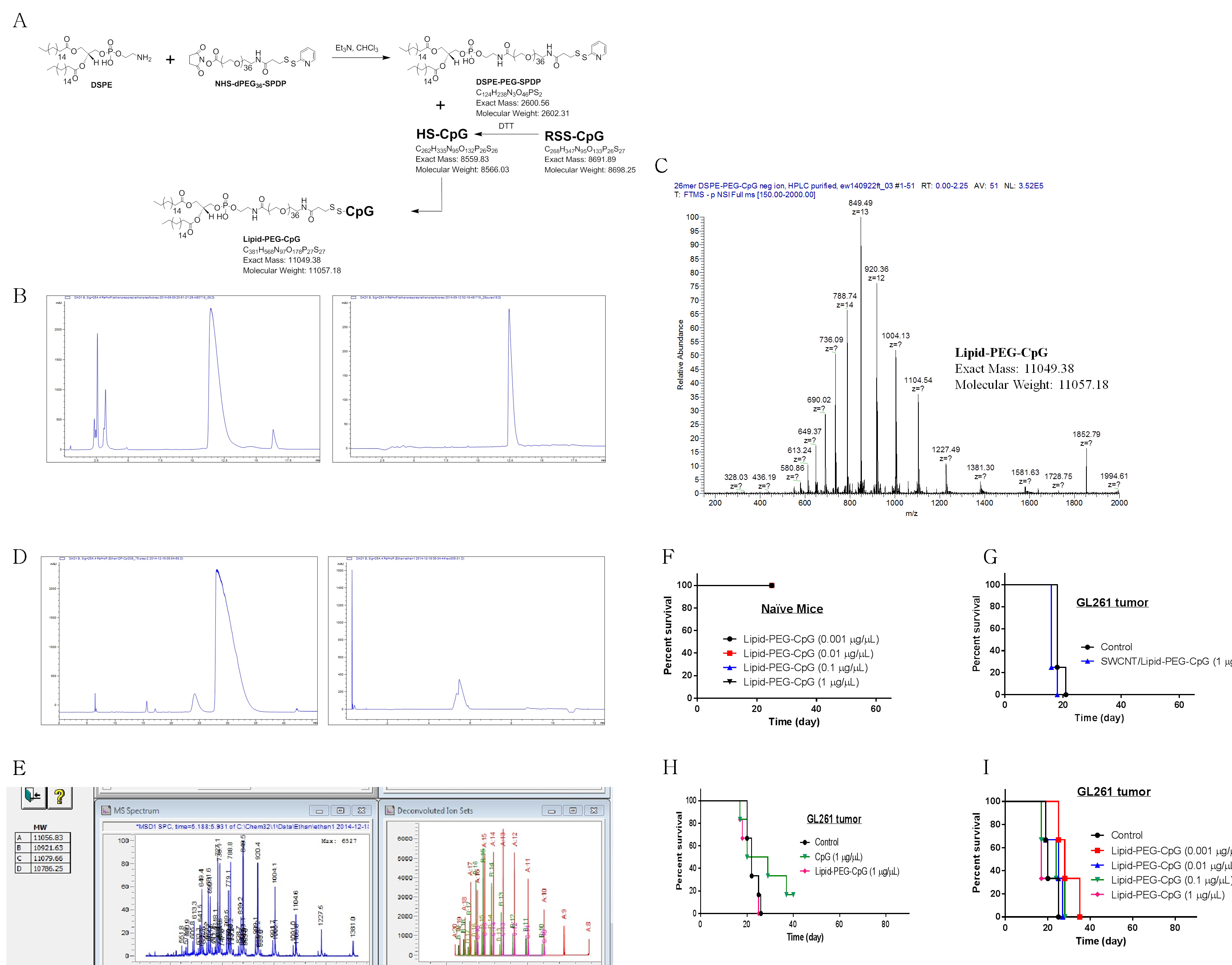

Supplement: S2 Fig — (A) Reaction scheme for the synthesis of Lipid-PEG-CpG. The PEG spacer used in this synthesis is discrete instead of the polydispersed PEG-2000 linker used in the original synthetic scheme. Therefore, the mass of the new Lipid-PEG-CpG in panel C is different than the theoretical mass in S1A Fig. (B) Absorbance trace at 254 nm (A254) of the prep HPLC purification (C18 column) of the first batch of Lipid-PEG-CpG (left panel) and the A254 trace (C18 column) of the combined purified fractions (right panel). (C) High-resolution mass spectrum of the Lipid-PEG-CpG isolated in B. (D) A254 trace of the second prep HPLC purification (phenyl column) of the second batch of Lipid-PEG-CpG (left panel) and the A254 trace (phenyl-hexyl column) of a representative purified fraction (right panel). (E) Low resolution mass spectrum from the LC-MS analysis of the peak in D. (F) Lipid-PEG-CpG is non-toxic. Naïve mice treated with various doses of Lipid-PEG-CpG (5 μL of the molar equivalent of 0.001 μg/μL to 1 μg/μL RSS-CpG) showed no signs of gross toxicity (n = 4). (G) GL261-tumor-bearing mice treated with SWCNT/Lipid-PEG-CpG showed no survival benefit compared to control (n = 4). (H) GL261-tumor-bearing mice treated with Lipid-PEG-CpG showed no survival benefit compared to RSS-CpG alone (the concentration of both samples was normalized to the molar equivalent of 1 μg/μL RSS-CpG) (n = 6). (I) GL261-tumor-bearing mice treated with various doses of Lipid-PEG-CpG (5 μL of the molar equivalent of 0.001 μg/μL to 1 μg/μL RSS-CpG) showed no survival benefit compared to control (n = 3). (TIF) [file pone.0148139.s002.tif]

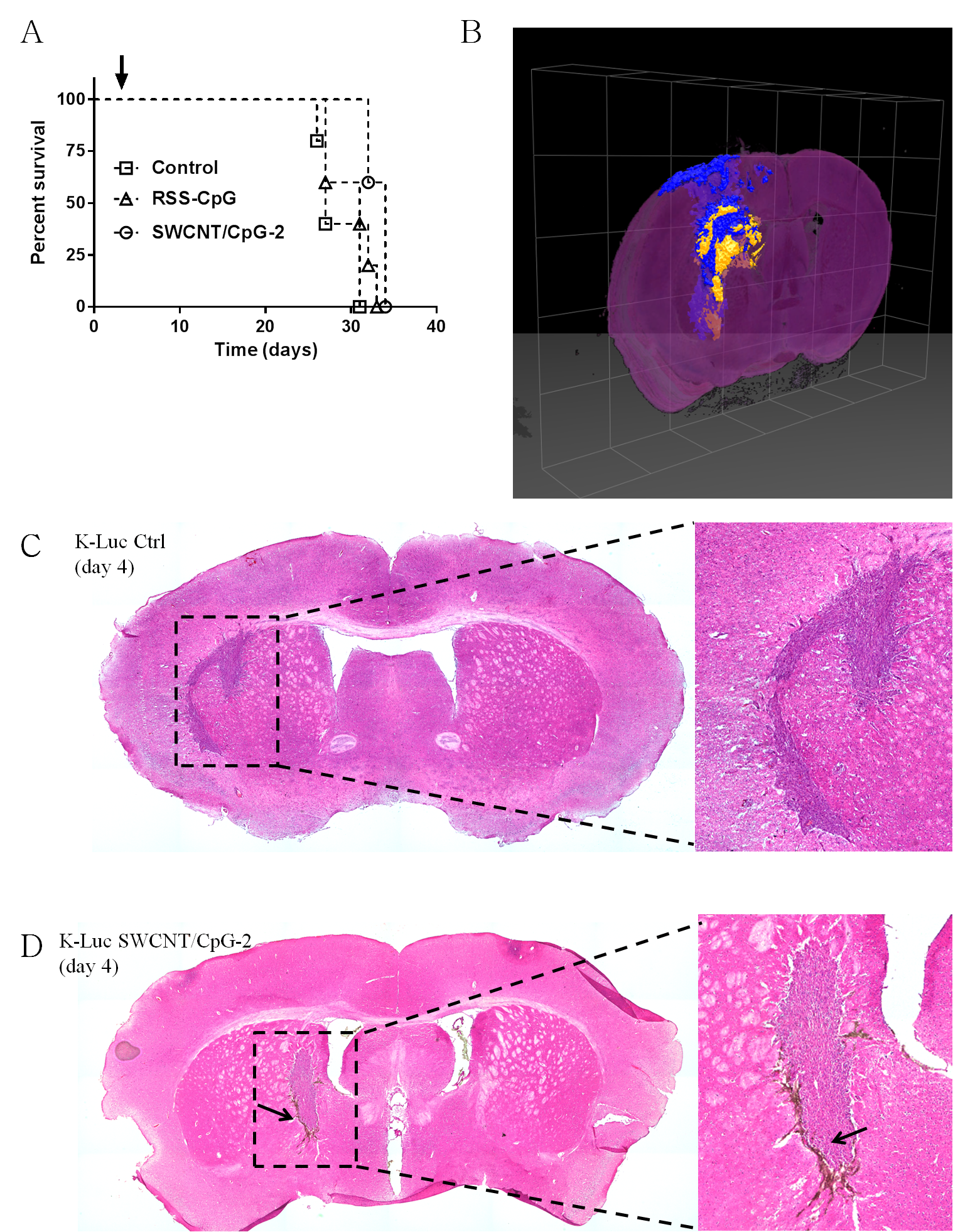

Supplement: S3 Fig — (A) Survival curve of tumor-bearing mice treated with a single injection (arrow) of either PBS, RSS-CpG, or SWCNT/CpG. Kaplan-Meier analysis showed that a single SWCNT/CpG treatment significantly extended survival when compared to both control and RSS-CpG (Log-rank test, P < 0.05, n = 5), but resulted in a lower median survival when compared to two treatments (34 days in S3A Fig vs 36 days and 39 days in Fig 2A and 2B). (B) 3D image of SWCNT/CpG and K-Luc tumor cells. K-Luc tumor cells (Blue) and SWCNT/CpG (Yellow). (C) H&E staining shows detectable tumor on Day 4 after K-Luc tumor implantation. (D) SWCNT/CpG treatment injected on Day 4 co-localizes with the tumor. The arrows indicate visible SWCNT accumulation (brown discoloration). (TIF) [file pone.0148139.s003.tif]

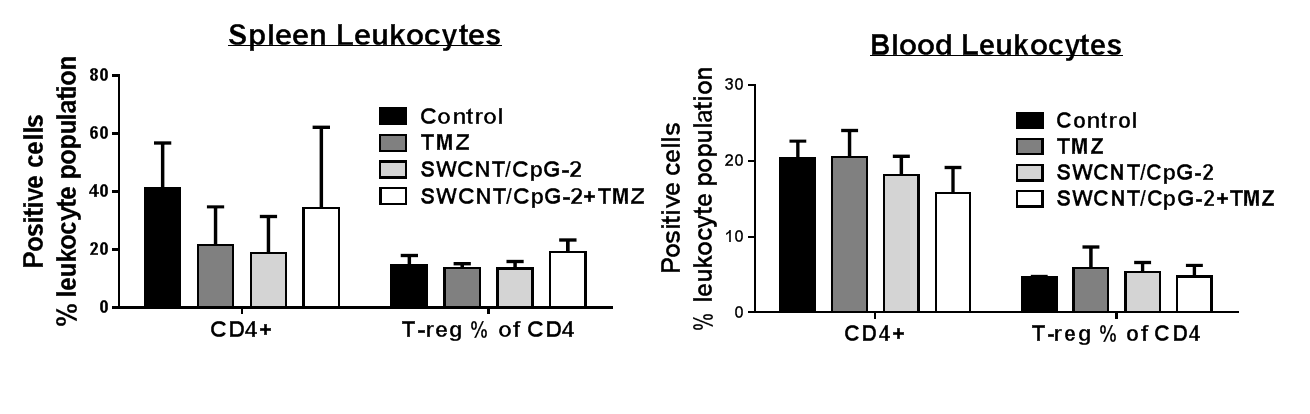

Supplement: S4 Fig — Frequency of CD4+ T cells and regulatory T cells (CD4+FoxP3+) in the spleen (left panel) and blood (right panel) was measured 10 days after treatment (14 days after tumor implantation) (n = 3 for TMZ group, n = 4 for other groups). (TIF) [file pone.0148139.s004.tif]
